# Late-onset severe hepatic sinusoidal obstruction syndrome in an allogeneic stem cell transplant recipient: A case report

**DOI:** 10.1097/MD.0000000000022927

**Published:** 2020-10-23

**Authors:** Yan-ling Sun, Ling-ling Liu, Yi He, Jing-wen Zhang, Rui-juan Wen, Qing Yuan, Xin Wang, Ruo-mi Guo, Xu-dong Li, Bing Long

**Affiliations:** a Department of Hematology; b Department of Blood Transfusion, The Third Affiliated Hospital, Sun Yat-sen University; c Xiancun Street Community Health Service Center of Tianhe District; d Department of Radiology, the Third Affiliated Hospital, Sun Yat-sen University, Guangzhou, China.

**Keywords:** hematopoietic stem cell transplantation, sinusoidal obstruction syndrome, thrombocytopenia

## Abstract

**Rationale:**

Hepatic sinusoidal obstruction syndrome (SOS) is a rare and potentially fatal complications after hematopoietic stem cell transplantation (HSCT). Most severe SOS result in multi-organ dysfunction and are associated with a high mortality rate (>80%).

**Patient concerns:**

A 31-year-old man was diagnosed with chronic myeloid leukemia blast crisis. He presented with severe thrombocytopenia on day 42 post-HSCT (on days +42), gradually developed with painful hepatomegaly, ascites, and weight gain.

**Diagnoses:**

The abdominal computerized tomography showed hepatomegaly, hepatic congestion, periportal edema, narrow hepatic vein, and ascites suggestive of SOS/hepatic vein occlusion. According to the EBMT revised diagnostic criteria, the patient was diagnosed as late-onset severe SOS.

**Interventions:**

Comprehensive treatment including low molecular weight heparin was initiated.

**Outcomes:**

The patient had good response with resolution of his hepatomegaly, increase of platelet, weight and transaminase loss after 4 weeks treatment.

**Lessons:**

In SOS patients with nonspecific clinical and biochemical findings, computerized tomography scans can be useful in differentiating SOS from other complications after HSCT. low molecular weight heparin is effective for the treatment of SOS.

## 1 Introduction

Hepatic sinusoidal obstruction syndrome (SOS), also known as hepatic vein occlusion (VOD), is one of the potentially devastating complications hematopoietic stem cell transplantation (HSCT). Most (75%–80%) of the patients with mild and moderate SOS can be relieved after 2 to 3 weeks of management, while the mortality of severe SOS is up to 80% to 90%.^[1]^ SOS usually develops within 21 days after HSCT, while few cases of late-onset SOS have been reported. It is characterized by post-sinusoidal portal hypertension results in a variety of symptoms including painful hepatomegaly, weight gain, fluid retention with ascites and jaundice. Here, we describe a HSCT recipient who developed late-onset severe SOS with nonspecific clinical and biochemical findings, finally diagnosed by computerized tomography (CT) scan.

## 2 Case report

A 31-year-old man was admitted to our hospital with thrombocytopenia on day 42 following unrelated donor allo-HSCT for chronic myeloid leukemia blast crisis (CML-BC). His history was significant for CML in chronic phase (CML-CP) diagnosed in July 2017, but progressed to CML-BC in May 2019 due to irregular imatinib treatment. He received dasatinib combined with induction chemotherapy (IA, idarubicin, and cytarabine) followed by one consolidation courses. He achieved a CR to induction, conditioned with Bu/Cy (busulfan and cyclophosphamide), and received a HSCT on September 10, 2019. He received antithymocyte globulin, cyclosporine, mycophenolate mofetil, and methotrexate for graft-versus-host disease (GVHD) prophylaxis, ursodeoxycholic acid, and Prostaglandin E1 for SOS prophylaxis.

He had no history of hepatitis and GVHD. Physical examination revealed a temperature of 37°C, mild anemia and petechiae of both legs. Initial investigations revealed a Hb level of 111 g/L (MCV of 101 fl), WBC 5.7 × 10^9^/L (granulocytes 58%, lymphocytes 32%), platelets 8 × 10^9^/L, AST of 34U/L, ALT of 20U/L, serum albumin of 34 g/L, direct bilirubin of 3.6 μmol/L, indirect bilirubin of 4.7 μmol/L, creatine of 75 μmol/L. DNA quantitative results for CMV, EBV, and herpes virus were negative. Examination of the bone marrow aspiration showed suppression of platelet production by megakaryocytes, with normal erythropoiesis and myelopoiesis. No residual blasts were detected in the bone marrow by FACS analysis, and the BCR-ABL1/ABL transcripts was below 0.01%. The DNA genotype of the bone marrow were completely transformed to the donor's. He was initially empirically treated with thrombopoietin and low dose prednisolone with no response. Additionally, the patient's thrombocytopenia refractory to platelet transfusion was noted.

**Figure 1:**
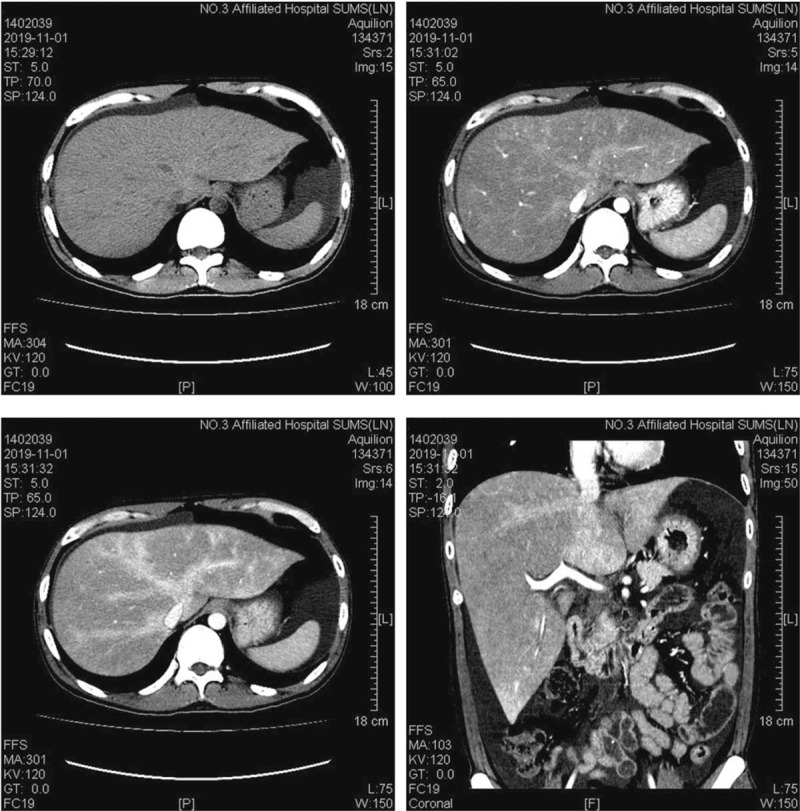
Abdominal computerized tomography scan showed hepatomegaly, hepatic congestion, periportal edema, narrow hepatic vein, and ascites suggestive of sinusoidal obstruction syndrome/hepatic vein occlusion.

During observation, he had complained of a dull pain and distention in the inferior sword area and right upper quadrant 1 week after hospitalization. Physical examination suggested palpable liver edge, ascites, and weight gain of 11 pounds (from 108 pounds–119 pounds). Further laboratory tests revealed persistent thrombocytopenia, moderate anemia, elevated levels of ALT (144U/L), AST (117U/L), direct bilirubin (8.5 μmol/L), and LDH (314U/L). Abdominal ultrasonography demonstrated hepatomegaly, ascites, and abnormal portal vein waveform. The abdominal CT showed hepatomegaly, hepatic congestion, periportal edema, narrow hepatic vein and ascites suggestive of SOS/VOD (Fig. 1). Due to painful hepatomegaly, ascites, weight gain >10%, and CT evidence of SOS/VOD, late onset severe SOS/VOD was diagnosed.

**Figure 2:**
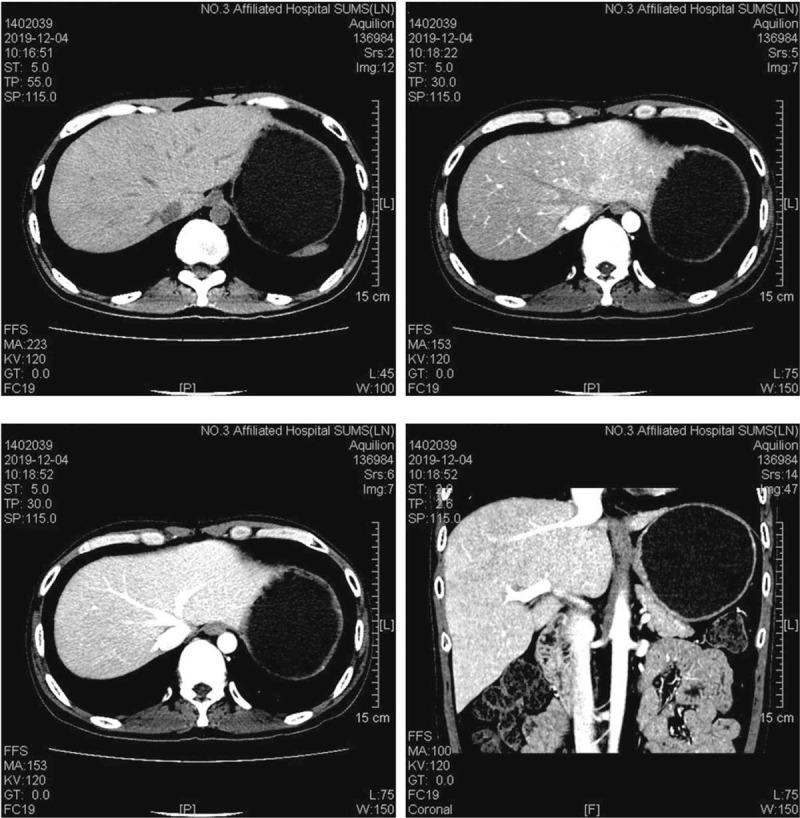
computerized tomography scan showed liver enlargement was decreased to normal, ascites, and hepatic congestion was improved after 4 weeks treatment.

**Figure 3:**
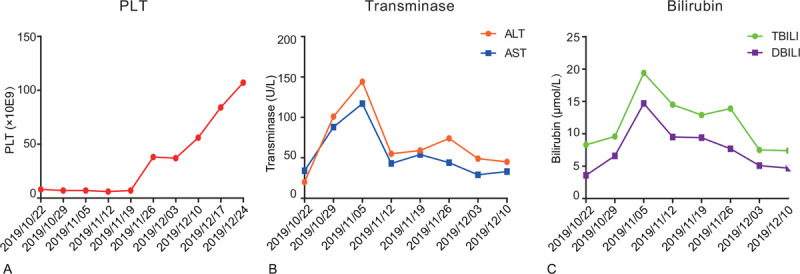
Laboratary data during the clinical course. (A) PLT (B) Transminase with ALT and AST (C) Bilirubin with total bilirubin and direct bilirubin.

He was treated with supportive care consisting of light diuresis for fluid management, cyclosporine was then replaced by mycophenolate mofetil. A treatment with ursodeoxycholic acid, Prostaglandin E1, and 0.5 mg/kg methylprednisolone was immediately initiated. Because of the defibrotide has not been approved in China, tissue plasminogen activator was not recommended due to the associated risk of hemorrhage, low molecular weight (LMWH) was started after discussion with the specialist hepatology unit. The patient had good response with resolution of his abdominal pain and weight loss. Liver enlargement was decreased to normal, ascites, and hepatic congestion was improved by CT scan after 4 weeks treatment (Fig. 2). He has been regularly followed-up in our outpatient clinic, has remained systemically well and his platelet have continued to gradually improve, serum bilirubin, and transaminase levels were decreased to normal (Fig. 3).

Informed consent for the collection of his medical history and blood samples was obtained in compliance with the Declaration of Helsinki and approved by the ethics committee of The Third Affiliated Hospital of Sun Yat-sen University. The patient provided informed consent for publication of the case.

## 3 Discussion

SOS has been described in patients who drank infusions made with plants containing pyrrolizidine alkaloids.^[2]^ It was also observed in relation to other pathogens such as alcohol, contraceptives, toxic oil, liver radiation, antineoplastic drugs. Since the first case of SOS following HCST reported in 1979, HSCT has proved to be the main cause of SOS.^[3]^ The specific pathogenesis of SOS is still unclear. Injury of sinusoidal endothelial cell and hepatocyte on zone 3 of hepatic lobule is considered to be the initial factor of SOS, which leads to hepatic venous outflow obstruction.^[4]^

The median incidence of SOS in HSCT patients reported was 13.7% (0%–62.3%).^[5]^ It is generally believed that the incidence is related to the experience of transplantation center, the risk factors of SOS and the diagnostic criteria. The morbidity is 10% to 15% after allo-HSCT with myeloablativecondition regimen, against <5% after auto-HSCT or allo-HSCT conditioned with reduced intensity pretreatment.^[6]^

Classical SOS usually develops within 21 days after HSCT, while those occurring beyond day 21 are so called late-onset SOS.^[7]^ Late-onset SOS is a rare disease with little literature reporting and the reason is still unclear. It is reported to be related to older age, busulphan-containing regimen, vancomycin treatment, quinolone group of antibiotics used for antimicrobial prophylaxis, and the use of ursodiol in transplantation.^[8]^ Ursodiol may inhibit the early evolution of SOS in patients with predisposing SOS risk factors, resulting in a delayed onset of SOS in some patients. Our patient received buslulphan, ofloxacin and ursodiol treatment, which may be the potential risk factor for late-onset SOS.

The classic diagnosis standard of SOS, Seattle^[9,10]^ and Bltimore^[11]^ criteria, are generally used to establish a clinical diagnosis. Both of them require that patients must be within 21 days after HCST to make the diagnosis of this complication, including the triad of painful hepatomegaly, jaundice, and fluid retention. However, our patient did not show the typical clinical features. He developed clinical signs beyond day 21 post-HSCT (on days +42), showed a severe thrombocytopenia with poor response to platelet transfusion, gradually developed with painful hepatomegaly, ascites, and weight gain, but no jaundice. It is necessary to differentiate it from acute GVHD, acute liver injury (drug or virus-induced), or transplantation related thrombotic microangiopathy. According to the revised diagnostic criteria which has been endorsed by the EBMT,^[7]^ hyperbilirubinemia is on longer mandatory and the criteria is less stringent for the diagnosis of late onset SOS. For our patient, hyperbilirubinemia never occurred throughout the course, he showed rapidly consumption of platelets as the initial manifestation, instead of edema, hepatomegaly, ascites, or jaundice. Peripheral thrombocytopenia with a rapid consumption of transfused platelets is frequently observed in patients with SOS, and it has been debated whether it should be included as a diagnostic criterion. However, this feature is difficult to evaluate during the pancytopenic phase after conditioning, and lack specificity, given the numerous causes of thrombocytopenia after HCT.

It should be noticed that hemodynamic and/or ultrasound evidence of SOS is mandatory in the diagnosis of late onset SOS, unless histologically proven SOS. However, ultrasonography was not helpful for our patient, which only showed hepatomegaly. Liver biopsy was not performed due to severe thrombocytopenia. Recently, given the diagnostics of key imaging features, other noninvasive imaging techniques, including CT, magnetic resonance imaging (MRI) and fluorodeoxyglucose positron emission tomography-computed tomography, have been widely used in this setting.^[12–14]^ In the early stage of SOS, there was no characteristic manifestation in US. CT has a high value in the diagnosis. For some patients, visualization of portosystemic collateral vessels with 3-dimensional spiral CTA is useful in the diagnosis.^[15]^ The abdominal CTA of our patient showed the typical signs of SOS. The normal CT scan showed non-uniform density of the liver parenchyma, hepatomegaly, and medium amount of ascites. Portal phase shows patchy low-density with a non-uniform decrease of hepatic parenchyma enhancement, ill-defined hepatic veins, and small branches.

Patients with mild SOS usually does not require treatment. Moderate disease can be treated with liquid balance for 2 days, if the symptoms still continuous or disease progresses, other treatment can be started. For severe disease, treatment should be started immediately. According to the BCSH/BSBMT guideline, defibrotide is recommended in the treatment of SOS in adults and children.^[16]^ The remission rate of defibrotide can be 50% to 55% and it is suggested to use early. In recent years, low dose rh- tissue plasminogen activator has been used to treat the disease and achieved high remission rate. In our patient, early intervention of supportive care and LMWH treatment improved the outcome.

## 4 Conclusions

Late-onset SOS should be considered in the differential diagnosis of HSCT recipients who present with unexplained ascites and hepatomegaly beyond day 21. In SOS patients with nonspecific clinical and biochemical findings, CT scans can be useful in differentiating SOS from other complications. LMWH is effective for the treatment of SOS.

## Acknowledgments

The authors would like to thank our patient for allowing us to write up his case and for his enthusiastic support throughout the process.

## Author contributions

**Conceptualization:** Yan-ling Sun, Yi He, Xu-dong Li, Bing Long.

**Data curation:** Yan-ling Sun, Ling-ling Liu, Jing-wen Zhang, Xin Wang, Bing Long.

**Funding acquisition:** Bing Long, Yan-ling Sun, Ling-ling Liu.

**Investigation:** Yan-ling Sun, Qing Yuan, Xin Wang, Xu-dong Li, Bing Long.

**Methodology:** Yan-ling Sun, Rui-juan Wen, Ruo-mi Guo, Bing Long.

**Project administration:** Yan-ling Sun, Bing Long.

**Supervision:** Bing Long.

**Writing – original draft:** Yan-ling Sun, Bing Long.

**Writing – review & editing:** Yan-ling Sun, Yi He, Jing-wen Zhang, Xu-dong Li, Bing Long.
